# Vision-Related Quality of Life in Patients With Keratoconus: A Nationwide Study in Saudi Arabia

**DOI:** 10.7759/cureus.35178

**Published:** 2023-02-19

**Authors:** Walaa Al-Dairi, Abdulaziz M Al Dehailan, Yazeed Alhammadi, Hussain I Aljohar, Faisal A Alhadi, Zeyad A Alhaboob, Ossama M Zakaria

**Affiliations:** 1 Surgery, King Faisal University, Al Ahsa, SAU; 2 College of Medicine, King Faisal University, Al Ahsa, SAU; 3 Pediatric Surgery, Suez Canal University, Ismailia, EGY; 4 Surgery/Pediatric Surgery, King Faisal University, Al Ahsa, SAU

**Keywords:** nei-vfq-25, saudi arabia, vision-related quality of life, nei-vfq, quality of life, keratoconus

## Abstract

Purpose: To evaluate the impact of keratoconus (KC) on quality of life and assess visual performance via the National Eye Institute Visual Functioning Questionnaire-25 (NEI-VFQ-25) in the Saudi population.

Patients and methods: A descriptive cross-sectional study was conducted using the NEI-VFQ-25 to evaluate the vision-related quality of life among previously diagnosed KC patients. An online questionnaire was used to distribute the validated survey through various social media networks. The data were extracted, reviewed, coded, and then analyzed using the Statistical Package for Social Sciences (SPSS) version 26 (IBM Corp., Armonk, NY).

Results: A total of 429 patients completed the questionnaire. The overall score of NEI-VFQ-25 was 58.6 (SD: 18.0). The visual performance was worse in females than males (with a score of 55.1), especially in patients aged less than 30 years. Visual function improved with the use of low-vision aids (spectacles and contact lenses) compared with those who did not use them.

Conclusion: Our study confirms the functional impairment in patients with KC, especially in females, patients aged less than 30 years, and those with no low-vision aids. Moreover, it suggests a significant role of these vision aids (spectacles and contact lenses) in improving the quality of life in patients with KC.

## Introduction

Keratoconus (KC) is a multifactorial ectatic corneal disease characterized by progressive corneal thinning and subsequent bulging of the corneal structure. This progression, if not halted, usually leads to the development of myopization and irregular astigmatism, resulting in impairment of visual acuity and quality of vision [1]. Typically, it begins in late childhood, clinically manifests in adolescence age, and persists till the third to fourth decade of life [2]. The prevalence of KC ranges from 0.05% in the United States to 18.7% in Saudi Arabia [3,4]. Depending on its severity, KC can be managed non-surgically by spectacles, lenses (either soft, rigid, or scleral), intrastromal corneal ring implants, or corneal collagen crosslinking. If still not effective, surgical management is usually performed (either lamellar or penetrating keratoplasty) [1,5,6]. In previous studies, it has been found that the management of choice in KC patients ultimately has a detrimental impact on their quality of life (QoL) [1,6,7].

The impact of chronic eye disease on daily activities can be measured by vision-related QoL, a good and measurable health outcome in patients with visual impairment [8]. The National Eye Institute Visual Functioning Questionnaire-25 (NEI-VFQ-25) has been found to be a validated instrument for measuring vision-related QoL [9]. This 25-item questionnaire has been used to assess patients’ QoL in a variety of eye-related disorders [10-14].

Evaluating the QoL in patients with KC serves as an insight into how the disease influences all aspects of their well-being and has become an important measure of disease management tailored to each patient. Therefore, this study aimed to explore the association between vision-related QoL and relevant sociodemographic and clinical variables in a group of patients with KC in Saudi Arabia, using the NEI-VFQ-25.

## Materials and methods

Study design and population

This descriptive cross-sectional study was conducted using the NEI-VFQ-25 to evaluate the QoL in Saudi patients with KC. The validated questionnaire was distributed among the largest online Saudi KC support group (available on Twitter and Telegram) with 1161 participants using Google Forms (Google, Mountain View, CA). This study included adults previously diagnosed with KC, who electronically signed a voluntary informed consent to participate in this study. All individuals under the age of 18 years or over the age of 60 years were excluded. All procedures performed were in accordance with the ethical standards of the Institutional Research Committee at King Faisal University (KFU) and with the 1964 Helsinki Declaration and its later amendments or comparable ethical standards. The data variables that were collected were age, gender, education level, residence region, and living location.

Questionnaire

The main theme variable was QoL related to vision and health obtained using NEI-VFQ-25 [15], a self-administered, vision-specific, patient-reported outcome measure that reports on real-world visual function and is globally validated for a variety of ocular conditions. The NEI-VFQ-25 mainly measures general health, visual acuity, eye pain, difficulty seeing near/far objects, and social functioning. The total score ranges from 0 to 100. Higher values ​​indicate better results [15]. The NEI-VFQ-25 has adequate validity and reliability, with α = 0.831 (95% CI: 0.735-0.904) in the entire questionnaire and >0.70 in all subscales except in “driving,” which, as in its official version, obtained lower reliability because it is an activity that not everyone performs, thus reducing the response rate [16].

Statistical analysis

Descriptive statistics of sample characteristics were calculated and presented as absolute frequencies and percentages for categorical variables and as mean and standard deviation (SD) for continuous variables. The normality of data was contrasted using the Kolmogorov-Smirnov test. Mann-Whitney or Kruskal-Wallis tests were used to assess associations between QoL and categorical variables related to health and vision. Two-tailed analysis with 0.05 was used as a cutoff for statistical significance. All data analyses were performed using the Statistical Package for Social Sciences (SPSS) version 26 (IBM Corp., Armonk, NY).

## Results

The present study consisted of 429 participants with the diagnosis of KC, who agreed to fill out our questionnaire. The socio-demographic characteristics of the patients are presented in Table 1. Among the participants, the most common age group was 30-40 years, with male gender predominance, i.e., nearly two-thirds (65.5%). A large majority of study participants mostly were bachelor’s degree holders (67.1%). Approximately 43.1% of the patients were living in the central region of Saudi Arabia and most were urban residents (86.9%).

**Table 1:** Socio-demographic characteristics of the patients (n = 429)

Study data	N (%)
Age group	
≤17 years	03 (0.70%)
18-25 years	56 (13.1%)
26-30 years	89 (20.7%)
31-35 years	106 (24.7%)
36-40 years	110 (25.6%)
>40 years	65 (15.2%)
Gender	
Male	281 (65.5%)
Female	148 (34.5%)
Educational level	
High school or below	95 (22.2%)
Bachelor’s degree	288 (67.1%)
Master’s degree or higher	46 (10.7%)
Residence region	
Northern Region	38 (08.9%)
Eastern Region	42 (09.8%)
Central Region	185 (43.1%)
Western Region	90 (21.0%)
Southern Region	74 (17.2%)
Living location	
Urban	373 (86.9%)
Rural	56 (13.1%)

Regarding the characteristics of KC patients in our study, more than one-third (35.4%) had a duration of KC of more than 15 years. The proportion of patients with a family history of KC was 33.3%. The commonly known type of correction was contact lens (50.8%) while the most common obstacles encountered when using contact lenses were recurrent inflamed, allergy, or dry eye disease (29.1%). Corneal cross-linking (CXL) has been the most frequent surgical procedure done for patients (31.5%). Regarding the level of satisfaction toward corneal transplant, patients were either satisfied (15.9%) or partially satisfied (18.4%) with the procedure. Of those who underwent CXL, 9.5% of them indicated that they repeated the procedure as it is required (Table 2).

**Table 2:** Characteristics of keratoconus patients (n = 429)

Variables	N (%)
Time since keratoconus diagnosis	
1 year or less	25 (05.8%)
1-5 years	88 (20.6%)
5-10 years	85 (19.8%)
10-15 years	79 (18.4%)
More than 15 years	152 (35.4%)
Family history of keratoconus	
Yes	143 (33.3%)
No/unknown	286 (66.7%)
Type of correction	
No optical correction	91 (21.2%)
Spectacles	95 (22.1%)
Contact lens	218 (50.8%)
What obstacles did you face with contact lenses?	
None	59 (13.8%)
Adaption obstacles	83 (19.3%)
Cost	114 (26.6%)
Fear	14 (03.3%)
Recurrent inflamed, allergic, dry eye	125 (29.1%)
Not interested in contact lens	34 (07.9%)
Surgical procedures	
None	175 (40.8%)
Corneal cross-linking (CXL)	135 (31.5%)
Intrastromal corneal ring (ICR)	41 (09.6%)
Implantable contact lenses	06 (01.4%)
Partial corneal transplant	19 (04.4%)
Total corneal transplant	53 (12.4%)
Have you been managed by corneal transplantation?	
No	170 (39.6%)
In one eye only	113 (26.3%)
In both eyes	146 (34.0%)
Level of satisfaction toward corneal transplantation	
Satisfied	68 (15.9%)
Partially Satisfied	79 (18.4%)
Not Satisfied	94 (21.9%)
The recovery period is not completed yet	188 (43.8%)
Was repeated corneal cross-linking (CXL) required if you had the first one already? (n = 232)	
Yes	22 (09.5%)
No	210 (90.5%)

**Figure 1:**
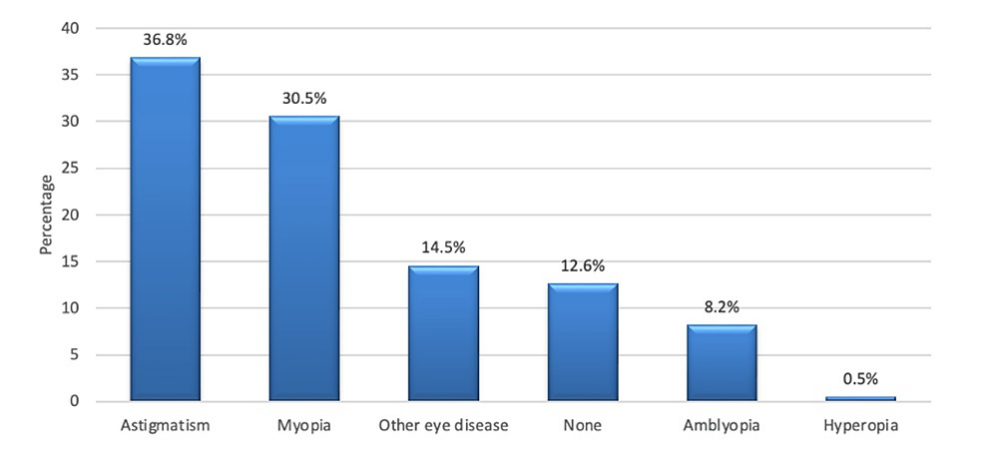
Other associated eye diseases

As shown in Figure 1, the most common associated eye diseases encountered in KC patients were astigmatism (36.8%) and myopia (30.5%).

**Figure 2:**
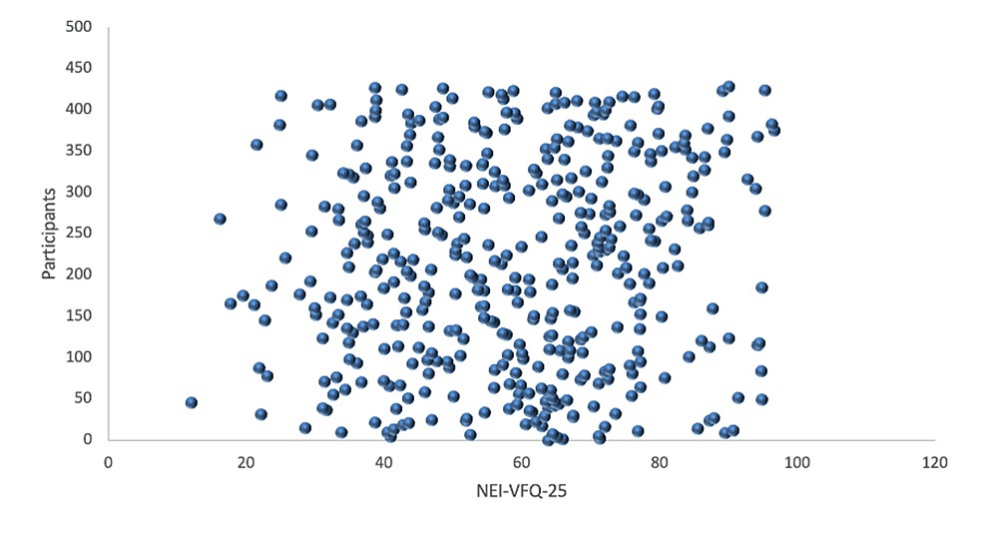
Distribution of total score of NEI-VFQ-25 NEI-VFQ-25: National Eye Institute Visual Functioning Questionnaire-25.

In Table 3, the median values of NEI-VFQ-25 subscales in descending order were shown as color vision (82.5), general vision (77.1), general health (73.5), social functioning (65.2), driving (60.4), near activities (58.7), dependency (56.1), ocular pain (55.8), distance activities (55.2), peripheral vision (53.9), role difficulties (52.8), and mental health (44.7). The overall median score of NEI-VFQ-25 was 58.6 (SD: 18). See the distribution of the overall median score of NEI-VFQ-25 in Figure 2.

**Table 3:** Descriptive statistics of NEI-VFQ-25 and its subscales (n = 429) NEI-VFQ-25: National Eye Institute Visual Functioning Questionnaire-25; VFQ: Visual Functioning Questionnaire.

VFQ subscale	Median ± SD
General health	73.5 ± 26.9
General vision	77.1 ± 22.9
Ocular pain	55.8 ± 21.9
Near activities	58.7 ± 24.9
Distance activities	55.2 ± 25.6
Vision specific	
Social functioning	65.2 ± 27.6
Mental health	44.7 ± 27.9
Role difficulties	52.8 ± 28.8
Dependency	56.1 ± 29.5
Driving	60.4 ± 23.1
Color vision	82.5 ± 24.7
Peripheral vision	53.9 ± 27.8
Overall score	58.6 ± 18.0

In Table 4, a statistical test revealed that a higher median mental health score and overall NEI-VFQ-25 score were associated with the older age group (>30 years). Male patients were more associated with a higher ocular pain median score and the overall NEI-VFQ-25 score. In addition, a higher median general vision score was more associated with patients who had a shorter duration of KC (≤10 years).

**Table 4:** Differences in median ranges of the subscales and total score of the NEI-VFQ-25 according to age, gender, and time since keratoconus diagnosis * p ≤ 0.05; ** p ≤ 0.01, p ≤ 0.001. NEI-VFQ-25: National Eye Institute Visual Functioning Questionnaire-25.

NEI-VFQ-25	Age group		Gender		Time of diagnosis	
	≤30 years	>30 years	Male	Female	≤10 years	>10 years
General health	69.4	75.3	74.6	71.5	72.3	74.6
General vision	75.2	77.9	76.1	78.9	79.7*	74.8*
Ocular pain	52.8	57.1	57.6*	52.4*	54.9	56.5
Near activities	55.6	59.9	59.9	56.3	57.3	59.8
Distance activities	51.4	56.8	55.8	53.8	53.6	56.5
Social functioning	63.2	66.1	66.6	62.5	63.9	66.4
Mental health	40.6*	46.5*	45.7	42.9	44.3	45.0
Role difficulties	51.6	53.3	52.7	53.0	52.5	53.1
Dependency	53.3	57.3	56.3	55.6	56.9	55.4
Driving	58.9	60.9	59.9	64.3	58.3	61.8
Color vision	83.6	82.1	81.9	83.6	83.9	81.3
Peripheral vision	54.0	53.9	53.4	55.1	54.3	53.7
Overall score	55.8*	59.9*	60.5**	55.1**	57.8	59.3

As shown in Table 5, correction with spectacles was more associated with higher median scores in ocular pain, social functioning, color vision, and the overall NEI-VFQ-25. Additionally, correction with contact lenses was associated with a higher median score in the general vision subscale.

**Table 5:** Differences in median ranges of the subscales and total score of the NEI-VFQ-25 according to the type of correction * p ≤ 0.05; ** p ≤ 0.01, p ≤ 0.001. NEI-VFQ-25: National Eye Institute Visual Functioning Questionnaire-25.

NEI-VFQ-25				
	Type of correction			
	No correction	Spectacles	Contact lens	
General health	71.9	73.9	74.3	
General vision	72.3	71.8	81.7**	
Ocular pain	50.4	64.2**	53.9	
Near activities	54.1	64.9	57.1	
Distance activities	53.5	59.9	53.7	
Social functioning	60.7	75.1**	62.9	
Mental health	38.1	51.8	42.9	
Role difficulties	48.2	60.7	50.3	
Dependency	52.9	64.5	52.3	
Driving	57.1	63.3	58.3	
Color vision	79.1	88.7*	80.7	
Peripheral vision	52.7	57.7	52.4	
Overall score	54.7	63.7**	57.5	

In our study, there were no significant differences in the median scores of the type of surgical procedures in all the subscales of NEI-VFQ-25 and its overall score (all p > 0.05) (Table 6).

**Table 6:** Differences in median ranges of the subscales and total score of the NEI-VFQ-25 according to surgical procedures NEI-VFQ-25: National Eye Institute Visual Functioning Questionnaire-25; CXL: corneal-cross linking; ICR: intrastromal corneal ring; ICL: implantable contact lenses; PCT: partial corneal transplant; TCT: total corneal transplant.

NEI-VFQ-25	Type of surgical procedures				
	CXL	ICR	ICL	PCT	TCT
General health	70.4	75.0	91.7	59.2	75.9
General vision	78.1	71.7	83.3	75.8	71.3
Ocular pain	55.3	60.9	54.2	51.3	54.7
Near activities	60.6	60.7	51.4	60.6	57.0
Distance activities	56.5	50.6	44.4	48.6	52.9
Social functioning	65.7	66.7	56.3	65.3	65.9
Mental health	46.6	47.4	34.4	38.5	43.9
Role difficulties	54.6	57.6	45.8	50.0	46.9
Dependency	56.5	60.6	61.1	46.9	54.6
Driving	63.4	60.8	58.3	49.4	56.0
Color vision	84.7	81.9	75.0	81.6	81.6
Peripheral vision	55.2	57.3	50.0	51.3	54.7
Overall score	59.3	60.4	58.0	54.5	57.3

Patients who have no other associated eye diseases were more associated with higher median scores in near activities, driving, and color vision while patients with concomitant hyperopia were more associated with a higher median score in mental health (Table 7).

**Table 7:** Differences in median ranges of the subscales and total score of the NEI-VFQ-25 according to other eye diseases * p ≤ 0.05; ** p ≤ 0.01, p ≤ 0.001. NEI-VFQ-25: National Eye Institute Visual Functioning Questionnaire-25.

NEI-VFQ-25	Other eye diseases					
	Astigmatism	Myopia	Hyperopia	Amblyopia	Other	None
General health	71.5	72.7	87.5	74.3	72.2	82.4
General vision	77.2	78.5	90.0	74.3	78.1	76.3
Ocular pain	55.9	54.9	50.0	52.1	54.4	59.3
Near activities	57.4	59.6	45.8	50.5	57.5	66.7*
Distance activities	55.5	54.4	50.0	47.5	51.7	60.7
Social functioning	66.4	63.9	50.0	58.5	62.9	68.4
Mental health	46.2	43.1	53.1*	35.5	44.2	49.4
Role difficulties	54.4	51.1	50.0	43.6	53.8	56.5
Dependency	56.7	54.9	37.5	48.3	55.2	63.4
Driving	60.5	58.5	41.7	50.6	57.8	70.4*
Color vision	81.5	83.5	75.0	73.6	84.4	85.4*
Peripheral vision	52.8	53.4	37.5	52.1	50.0	59.7
Overall score	58.8	58.1	53.9	53.7	56.7	63.3

## Discussion

Due to remarkable advancements in medical care, patient-centered approaches prioritize well-being and QoL when tailoring therapies. In the case of KC, a known cause of social burden is early visual impairment and outcome unpredictability. Previous studies have shown a significant decrease in the QoL for these patients [17]. Identifying the factors linked to this decrease might aid in the development of action protocols aimed at not only lowering or eradicating KC symptoms but also enhancing the QoL and well-being of those affected. In our study, the total score was 58.6 ± 18.0. The highest NEI-VFQ-25 subscale score was attributed to color vision (82.5 ± 24.7) and the lowest scores were related to mental health (44.7 ± 27.9). Comparing our results with previous studies examining the impact of KC on vision-related QoL, we found that our participants’ overall score was lower than French [18], Iranian [19], and Turkish KC patients [9]. In addition, our study scores were lower than the Collaborative Longitudinal Evaluation of Keratoconus (CLEK) scores, except for general vision [17]. Compared to other chronic ocular diseases, the scores of near and distance activities in our study differ from the scores of glaucoma patients [11], but they are close to previously reported scores of age-related macular degeneration [13]. Given the fact that a higher median mental health score and overall NEI-VFQ-25 score were observed in the older age group (>30 years), this could mean that as the KC patients get older, they will develop perceptual adaptation toward the disease. This suggests that clinical indicators like visual acuity are not the only ones that influence patients' perceptions of their illness. Adopting patient-reported outcome measures also provides more detailed and useful information to lessen the KC burden.

Socio-demographic data and time since KC diagnosis exhibited an association with NEI-VFQ-25 scores. A higher median general vision score was more associated with patients who had a shorter duration of KC (≤10 years). In accordance with Gothwal et al., patients with KC disease duration of more than three years showed worse ratings on both the functional and emotional well-being measures [20]. Consistent with our findings, a previous study showed a considerable improvement in the vision-related QoL in KC patients equipped with implantable contact lenses (ICL), with a 19.5-point increase in the total score [1]. It is interesting to note that, as revealed in participant responses, there were no significant differences in the median scores of the type of surgical procedures in all the subscales of NEI-VFQ-25 and its overall score (all p > 0.05).

In our study, asking about the association between driving and the QoL in patients with KC is the main weakness since not all the participants drive, thus influencing the response rate.

## Conclusions

Our patients with KC had mental, visual, physical, and social impairment in their QoL. This perceived shortcoming was in excess compared with their clinical measures that confirm a previous theory about the disproportionate social burden of KC. Our findings showed that female patients, patients aged less than 30 years, and those with no low vision aids were associated with lower QoL scores using the NEI-VFQ-25. Strategies to improve the QoL in KC patients are warranted to maintain their QoL. Finally, our results provided evidence for the validity and reliability of the Saudi version of the NEI-VFQ-25, and potentially applicable in further investigations.
